# Hantavirus and Arenavirus Antibodies in Persons with Occupational Rodent Exposure, North America

**DOI:** 10.3201/eid1304.061509

**Published:** 2007-04

**Authors:** Charles F. Fulhorst, Mary Louise Milazzo, Lori R. Armstrong, James E. Childs, Pierre E. Rollin, Rima Khabbaz, C.J. Peters, Thomas G. Ksiazek

**Affiliations:** * University of Texas Medical Branch, Galveston, Texas, USA; † Centers for Disease Control and Prevention, Atlanta, Georgia, USA; 1 Current affiliation: Yale University School of Medicine, New Haven, Connecticut, USA; 2 Current affiliation: University of Texas Medical Branch, Galveston, Texas, USA

**Keywords:** Hantavirus, Sin Nombre virus, arenavirus, Bear Canyon virus, Tamiami virus, Whitewater Arroyo virus, lymphocytic choriomeningitis virus, research

## Abstract

Rodents are the principal hosts of Sin Nombre virus, 4 other hantaviruses known to cause hantavirus pulmonary syndrome in North America, and the 3 North American arenaviruses. Serum samples from 757 persons who had worked with rodents in North America and handled neotomine or sigmodontine rodents were tested for antibodies against Sin Nombre virus, Whitewater Arroyo virus, Guanarito virus, and lymphocytic choriomeningitis virus. Antibodies against Sin Nombre virus were found in 4 persons, against Whitewater Arroyo virus or Guanarito virus in 2 persons, and against lymphocytic choriomeningitis virus in none. These results suggest that risk for infection with hantaviruses or arenaviruses usually is low in persons whose occupations entail close physical contact with neotomine or sigmodontine rodents in North America.

## Abstract 2

Risk for infection was low among those who handled neotomine or sigmodontine rodents on the job.

Hantavirus pulmonary syndrome (HPS) is a frequently fatal rodentborne viral zoonosis. Seven species in the virus family *Bunyaviridae*, genus *Hantavirus* (*1*), have been causally associated with HPS: *Sin Nombre virus* (SNV), *New York virus* (NYV), *Black Creek Canal* *virus* (BCCV), *Bayou* *virus* (BAYV), and *Choclo virus* (CHOV) in North America (*2**–**6*), and *Andes virus* (ANDV) and *Laguna Negra virus* (LANV) in South America (*7**,**8*).

The virus family *Arenaviridae*, genus *Arenavirus*, includes 3 North American species and 14 South American species (*9*). The North American species are *Bear Canyon virus* (BCNV), *Tamiami virus* (TAMV), and *Whitewater Arroyo virus* (WWAV). The South American species include *Guanarito virus* (GTOV), *Junin virus* (JUNV), *Machupo virus* (MACV), and *Sabiá virus* (SABV). These 4 South American species have been causally associated with severe human disease in Venezuela, Argentina, Bolivia, and Brazil, respectively (*10*). The human health importance of the North American arenavirus species has not been rigorously investigated.

Specific members of the subfamilies Neotominae and Sigmodontinae in the rodent family Cricetidae (*11*) are the principal hosts (reservoirs) of the hantaviruses known to cause HPS in North America and the 3 North American arenaviruses. For example, principal hosts and their respective viruses include: the deer mouse (*Peromyscus maniculatus*) in Canada and the western United States, SNV (*12**,**13*); the white-footed mouse (*Peromyscus leucopus*) in the northeastern United States, NYV (*3*); the hispid cotton rat (*Sigmodon hispidus*) in Florida, BCCV and TAMV (*4**,**14*); the marsh rice rat (*Oryzomys palustris*) in the southeastern United States, BAYV (*15**–**17*); the fulvous colilargo (*Oligoryzomys fulvescens*) in Panama, CHOV (*6*); the California mouse (*Peromyscus californicus*) in California, BCNV (*18*); and the white-throated woodrat (*Neotoma albigula*) in New Mexico, WWAV (*19*). *P. maniculatus, P. leucopus*, *P. californicus*, and *N. albigula* are members of the Neotominae and *S. hispidus*, *O. palustris*, and *O. fulvescens* are members of the Sigmodontinae (*11*).

It is assumed that humans usually become infected with hantaviruses and arenaviruses by inhalation of aerosolized droplets of urine, saliva, or respiratory secretions from infected rodents. Other means of infection include, but are not limited to, inhalation of dust or other organic matter contaminated with infectious virus and contact of infectious materials with mucous membranes.

The purpose of this study was to assess the risk for hantavirus and arenavirus infections among persons who work in North America and have close physical contact with neotomine rodents or sigmodontine rodents through their occupations. These persons include mammalogists, wildlife biologists, scientists whose research concerns the ecology of rodent-borne zoonoses, and pest control operators.

## Materials and Methods

### Study Population

The persons in this study were participants in a survey conducted in 1994 by the Centers for Disease Control and Prevention (CDC). The primary objective of the survey was to assess the risk for hantavirus infections in persons whose occupations expose them to rodents. Participation in the survey was voluntary and entailed completion of a self-administered questionnaire and donation of a small volume of venous blood. Most of the 995 participants were enrolled at 1 of the following: American Society of Mammalogists meeting (Washington, DC, 1994), Wildlife Disease Association meeting (Pacific Grove, California, 1994), Southwestern Association of Naturalists meeting (Emporia, Kansas, 1994), Wildlife Society meeting (Wenatchee, Washington, 1994), 16th Vertebrate Pest Conference (Santa Clara, California, 1994), and Colorado Pest Control Meeting (Denver, Colorado, 1994). The other participants mailed their completed questionnaires and serum samples directly to CDC.

The questionnaire included detailed questions about previous exposure to rodents, use of personal protective equipment to minimize exposure to rodent excretions and secretions, and any previous occurrence of a severe febrile illness that included shortness of breath. The lifetime number of rodents handled by a person was measured categorically: I (1–99), II (100–499), III (500–999), IV (1,000–9,999), V (10,000–49,999), and VI (≥50,000). Use of gloves, protective masks equipped with high efficiency particulate air (HEPA) filters, and protective eyewear also was measured categorically: always (>90% of the time), usually (50%–90% of the time), sometimes (10%–49% of the time), seldom (<10% of the time), or never.

This study was restricted to the 757 participants in the CDC survey who had a history of exposure to rodents in North America and a history of occupational exposure to deer mice, white-footed mice, California mice, woodrats (*Neotoma* spp.), other neotomine rodents, cotton rats (*Sigmodon* spp.), oryzomyine rodents (*Oryzomys* spp. or *Oligoryzomys* spp.), or other sigmodontine rodents. Of the persons included in the study, 699 had worked with rodents only in North America. The 58 others had worked with rodents in North America and in South America. The geographic distribution of exposure to rodents in North America was Canada (n = 36), Alaska (n = 8), the contiguous United States or District of Columbia (n = 726, Table 1), Mexico (n = 91), Guatemala (n = 8), Belize (n = 3), Honduras (n = 3), Costa Rica (n = 21), Nicaragua (n = 4), and Panama (n = 8). Of the persons included in the study, 468 (61.8%) had worked with rodents in >1 state within the contiguous United States.

**Table 1:** Occupational exposure of 726 persons to sigmodontine or neotomine rodents within the contiguous United States

State	No. persons exposed*
Alabama	16†
Arkansas	33
Arizona	92†‡
California	178†‡
Colorado	128†‡
Connecticut	19
Delaware	1
Florida	17†‡
Georgia	15
Idaho	21†
Illinois	38†
Indiana	26†
Iowa	17†
Kansas	90†
Kentucky	6
Louisiana	11†
Maine	21
Massachusetts	17
Maryland	27
Michigan	38
Minnesota	21†
Mississippi	6
Missouri	36
Montana	21†
Nebraska	25†
Nevada	39†
New Hampshire	6
New Jersey	9
New Mexico	118†‡
New York	46†
North Carolina	15†
North Dakota	9†
Ohio	25
Oklahoma	41†‡
Oregon	45†
Pennsylvania	45†
Rhode Island	5
South Carolina	16
South Dakota	11†
Tennessee	23
Texas	112†‡
Utah	67†‡
Vermont	6†
Virginia	49†
Washington	73†
West Virginia	11†
Wisconsin	22†
Wyoming	41†
District of Columbia	2

*No. persons who worked with rodents in the state or in the District of Columbia. Of the persons in the study, 468 had worked with rodents in >1 state within the contiguous United States. †States that have reported ≥1 case of hantavirus pulmonary syndrome to the Centers for Disease Control and Prevention through September 19, 2006 (see www.cdc.gov/ncidod/diseases/hanta/hps/noframes/epislides /episl7.htm). ‡States in which neotomine or sigmodontine rodents are known to be naturally associated with Bear Canyon virus, Tamiami virus, or Whitewater Arroyo virus.

Persons included in the study had worked with rodents from 1 month to 65 years (mean 12.5 years). The total number of rodents handled by any 1 person ranged from category I (1–99) to category VI (≥50,000); the median was IV (1,000–9,999). Of the 757 persons in the study, 751 (99.2%) had handled deer mice, white-footed mice, cotton rats, oryzomymine rodents, California mice, or woodrats (Table 2).

**Table 2:** Work-related exposure of 757 persons to neotomine and sigmodontine rodents

No. persons*	Rodent†	Exposure‡	
		Range	Median
695	Deer mouse (*Peromyscus maniculatus*)	I–VI	II
487	White-footed mouse (*P. leucopus*)	I–VI	II
34	California mouse (*P. californicus*)	I–III	I
456	Woodrat (*Neotoma* spp.)	I–V	I
392	Other neotomine rodents	I–IV	I
511	Cotton rat (*Sigmodon* spp.)	I–VI	I
51	Oryzomyine rodents	I–IV	I

*No. persons who self-reported occupational exposure to rodents. †Other neotomine rodents included the pygmy mouse (*Baiomys* spp.), Texas mouse (*Peromyscus attwateri*), brush mouse (*P. boylii*), canyon mouse (*P. crinitus*), Zacatecan deer mouse (*P. difficilis*), cactus mouse (*P. eremicus*), Florida mouse (*P. floridanus*), cotton mouse (*P. gossypinus*), northern rock mouse (*P. nasutus*), golden mouse (*P. nuttalis*), white-ankled mouse (*P. pectoralis*), oldfield mouse (*P. polionotus*), pinyon mouse (*P. truei*), and western harvest mouse (*Reithrodontomys megalotis*) and other harvest mice (*Reithrodontomys* spp.). Oryzomyine rodents included the marsh rice rat (*Oryzomys palustris*), other oryzomine rodents (*Oryzomys* spp.), and oligoryzomine rodents (*Oligoryzomys* spp.). ‡The number of rodents handled by a study subject was categorized as follows: I, 1–99; II, 100–499; III, 500–999; IV, 1,000–9,999; V, 10,000–49,999; VI, >50,000.

HPS was first recognized as a clinical entity in 1993 in the southwestern United States (*20*). From March 1, 1993, through September 19, 2006, a total of 453 laboratory-confirmed HPS cases were reported to CDC from the contiguous United States (www.cdc.gov/ncidod/diseases/hanta/hps/noframes/epislides/episl7.htm). Of these, 259 (57.2%) were reported from 6 states in the southwestern United States: Colorado (n = 51), New Mexico (n = 71), Utah (n = 25), Arizona (n = 49), Nevada (n = 18), and California (n = 45). Sin Nombre virus is the only virus known to cause HPS in these 6 states. In this study, 387 (51.1%) persons had worked with rodents in Colorado (n = 124), New Mexico (n = 111), Utah (n = 65), Arizona (n = 90), Nevada (n = 33), or California (n = 169) and had handled deer mice. The total number of deer mice handled by persons in this group ranged from I (1–99) to VI (≥50,000); the median was II (100–499).

The geographic range of BAYV includes Georgia, Louisiana, and Texas (*5**,**15**–**17*), BCCV has been found only in Florida (*4*), and the geographic range of NYV includes New York, Pennsylvania, and Rhode Island (*3**,**21**,**22*). In this study, 22 persons had worked with rodents in Georgia (n = 1), Louisiana (n = 2), or Texas (n = 20) and handled oryzomyine rodents. The total number of oryzomyine rodents handled by persons in this group ranged from I (1–99) to IV (1,000–9,999); the median was I (1–99). Fourteen persons had worked with rodents in Florida and handled cotton rats. The total number of cotton rats handled by persons in this group ranged from I (1–99) to IV (1,000–9,999); the median was II (100–499). Eighty-one persons had worked with rodents in New York (n = 45), Pennsylvania (n = 42), or Rhode Island (n = 5) and handled white-footed mice. The total number of white-footed mice handled by persons in this group ranged from I (1–99) to V (10,000–49,999); the median was II (100–499).

BCNV virus has been found only in California (*18*) and TAMV only in Florida (*14*); the geographic range of WWAV and other arenaviruses naturally associated with woodrats (*Neotoma* spp.) includes Arizona, California, Colorado, New Mexico, Oklahoma, Utah, and Texas (*19**,**23**–**27*). In this study, 31 persons had worked with rodents in California and handled California mice (*P. californicus*). The total number of California mice handled by persons in this group ranged from I (1–99) to III (500–999); the median was I (1–99). As indicated previously, 14 persons had worked with rodents in Florida and handled cotton rats. Three hundred and thirty-three persons had worked with rodents in Arizona (n = 87), California (n = 130), Colorado (n = 76), New Mexico (n = 101), Oklahoma (n = 40), Utah (n = 59), or Texas (n = 101) and handled woodrats. The total number of woodrats handled by persons in this group ranged from I (1–99) to V (10,000–49,999); the median was I (1–99).

*Lymphocytic choriomeningitis virus* (LCMV) is the only Old World arenavirus species that is enzootic in North America. The house mouse (*Mus musculus*) is a member of the subfamily Murinae, family Muridae (*11*) and the principal host of LCMV. In this study, 526 (69.5%) persons had worked with house mice. The total number of house mice handled by persons in this group ranged from I (1–99) to VI (>50,000); the median was I (1–99).

Of the 757 persons in this study, 735 (97.1%) had worked with rodents before the discovery of HPS in 1993; during that time, 504 (68.6%) of them never or infrequently wore personal protective equipment (gloves, a protective mask equipped with HEPA filters, and protective eyewear) when handling rodents. In contrast, only 267 (36.3%) of these 735 persons never or infrequently wore personal protective equipment when handling rodents after the discovery of HPS. Use of personal protective equipment by the other persons in the study both before and after the discovery of HPS depended on the type of equipment.

All unique identifying information was removed from the serum samples before they were tested for antibodies. Furthermore, all unique identifying information was removed from the computer (electronic) records before analysis of the demographic and serologic data.

### Antibody Assays

We tested the serum samples for immunoglobulin G (IgG) against SNV, WWAV, GTOV, and LCMV by using ELISA, as described (*24**,**28*). The SNV antigen was an *Escherichia coli*–expressed recombinant SNV nucleocapsid protein that is highly cross-reactive with other neotomine rodent-associated hantaviruses and with sigmodontine rodent-associated hantaviruses in the ELISA used in this study (T.G. Ksiazek, unpub. data). The control (comparison) antigen for the SNV IgG ELISA was an *E. coli*–expressed recombinant protein that is antigenically unrelated to the SNV nucleocapsid protein. The arenavirus antigens were detergent lysates of Vero E6 cells infected with WWAV strain AV 9310135, GTOV strain INH-95551, or LCMV strain Armstrong. WWAV is highly cross-reactive with BCNV and TAMV in the ELISA used in this study (M.L. Milazzo, unpub. data). Collectively, WWAV, GTOV, and LCMV represent the 3 major antigen groups in the family *Arenaviridae*, as defined by ELISA (*19*). The control antigens for the arenavirus IgG assays were detergent lysates of uninfected Vero E6 cells. The working concentrations of the SNV, GTOV, and LCMV antigens and the corresponding control antigens were determined by checkerboard titration against convalescent-phase serum samples from humans infected with SNV, GTOV, and LCMV, respectively. The working concentrations of the WWAV antigen and the corresponding control antigen were determined by checkerboard titration against a mouse ascitic fluid against WWAV strain AV 9310135. Serial 4-fold dilutions (from 1:100 through 1:6,400) of each serum sample were tested against the 4 test antigens and 4 control antigens. Antibody bound to antigen was detected by using a goat anti-human IgG (gamma chain–specific) peroxidase conjugate (Kirkegaard and Perry Laboratories, Gaithersburg, MD, USA). Optical densities (OD) at 405 nm (reference = 490 nm) were measured with a Dynex MRX II microplate reader (Dynatech Industries, Inc., McLean, VA, USA). The adjusted OD (AOD) of a serum-antigen reaction was the OD of the well coated with the test antigen minus the OD of the well coated with the control antigen. A sample was considered positive if the AOD at 1:100 was ≥0.200, the AOD at 1:400 was ≥0.200, and the sum of the AODs for the series of 4-fold dilutions (from 1:100 through 1:6,400) was ≥0.900. These criteria for positivity were based on the results of previous work with the test antigens and control antigens. The antibody titer of a positive sample was the reciprocal of the highest dilution of that sample for which the AOD was ≥0.200.

## Results

Antibodies against SNV were detected in 4 (0.5%) of the 757 persons in the study. Antibody titers were 1,600 in 2 persons and ≥6,400 in the other 2 persons. The total years worked with rodents and the lifetime number of rodents handled by the 4 antibody-positive persons were 9.0–30.0 (mean 21.3) and IV (1,000–9,999) to V (10,000–49,999), respectively. Two of the antibody-positive persons had worked with rodents only within the contiguous United States (specifically Arkansas, Arizona, Colorado, Iowa, Kansas, Michigan, Oklahoma, South Carolina, and/or Texas), 1 had worked with rodents in Arizona, Colorado, New Mexico, Utah, Texas, and Mexico, and 1 had worked with rodents in Michigan, Pennsylvania, Mexico, Costa Rica, and Argentina. All 4 antibody-positive persons had handled deer mice, white-footed mice, other neotomine rodents, cotton rats, and other sigmodontine rodents. Those who had worked in South Carolina or Argentina also had handled oryzomyine rodents. All 4 antibody-positive persons reported that they had never worn a protective mask or protective eyewear when handling rodents before the discovery of HPS. One reported having been hospitalized for an illness characterized by fever, headache, and severe shortness of breath (symptoms suggestive of HPS). This person had worked with rodents only within the contiguous United States.

Antibodies against WWAV or GTOV were detected in 2 (0.3%) of the 757 persons in the study. Antibodies against WWAV (antibody titer = 1,600) but not GTOV were detected in a person who had worked with rodents in Texas and Wisconsin and handled woodrats, other neotomine rodents, and sigmodontine rodents. Antibodies against GTOV (antibody titer = 1,600) but not WWAV were detected in a person who had worked with rodents in Pennsylvania, Utah, and Wyoming and handled white-footed mice, other neotomine rodents, cotton rats, and other sigmodontine rodents. The lifetime number of rodents handled by the 2 antibody-positive persons were III (500–999) and IV (1,000–9,999), respectively. Both reported that they had never worn a protective mask or protective eyewear when handling rodents before the discovery of HPS. Antibodies against LCMV were found in none of the 757 persons in this study.

## Discussion

Previously published studies found no antibodies against SNV in 583 persons who worked in Arizona or New Mexico in occupations that potentially exposed them to rodents or rodent droppings (*29**,**30*) and no antibodies against SNV or WWAV in 72 persons in California whose occupations entailed close physical contact with rodents (*31*). Limited seroprevalence studies found antibodies against LCMV in up to 5.1% of healthy persons in the United States (*32**,**33*). If one discounts fatal infections and assumes that IgG against SNV and other hantaviruses is measurable many years after recovery from infection, the results of this study indicate that the risk for infection with hantaviruses usually is low in persons whose occupations entail close physical contact with neotomine rodents or sigmodontine rodents in North America. Similarly, the study results indicate that the risk for infection with arenaviruses usually is low in persons whose occupations entail close physical contact with neotomine rodents or sigmodontine rodents in North America

Some hantaviruses and arenaviruses appear to be restricted to small areas within the geographic ranges of the rodent species that serve as their natural reservoirs. For example, BCCV and TAMV have been found only in southern Florida (*4**,**14*), yet the geographic range of *S. hispidus* extends from Arizona, Nebraska, and Virginia through northeastern Mexico (*11*). Furthermore, the prevalence of infected rodents can vary widely even in a small area (*23**,**34*). Thus, the low prevalence of antibodies against SNV and against the arenaviruses included in this study could be because few of the rodents handled by the 757 persons in the study were infected with a hantavirus or arenavirus. Other explanations for the low prevalence of antibodies against SNV, WWAV, GTOV, and LCMV in this study are because the circumstances under which or the manner in which the rodents were handled did not favor rodent-to-human virus transmission or because tissues, secretions, and excretions from infected rodents are not highly infectious to humans.

Antibodies against SNV were detected in 3 (0.8%) of the 387 persons in this study who had worked with rodents in Colorado, New Mexico, Utah, Arizona, Nevada, or California and who had handled deer mice. Antibodies to SNV also were detected in 1 (1.2%) of the 81 persons who had worked in New York, Pennsylvania, or Rhode Island and who had handled white-footed mice. The antibodies against SNV in the 3 antibody-positive persons who had worked in the southwestern United States could be a consequence of infection with SNV. The antibodies against SNV in the person who had worked in Pennsylvania could be a result of infection with NYV.

Of the 453 laboratory-confirmed HPS cases mentioned previously, 160 (35.3%) were fatal. Together, the high case-fatality ratio of HPS in North America, the lack of a vaccine against HPS, and the lack of a specific therapy for HPS should motivate persons to minimize their risk for infection while working in the field, classroom, or laboratory with rodents potentially infected with hantaviruses, especially those viruses known to cause HPS. Published guidelines for safely working with rodents potentially infected with hantaviruses include using protective gloves, respirators fitted with HEPA filters, and protective eyewear (*35*). None of the 4 persons in the study who were antibody-positive against SNV had worn gloves, masks, or protective eyewear when handling rodents before the discovery of HPS.

The use of personal protective equipment in the field may seem cumbersome. However, 2 recent HPS cases, 1 fatal, underscore the need to use appropriate personal protective equipment and follow recommended safety procedures when working with rodents potentially infected with hantaviruses that have been causally associated with HPS. The fatal case was in a graduate student who was studying the effects of forest management practices on small mammal populations in West Virginia (*36*). The nonfatal case was in a field technician who was trapping rodents as part of a forest health study in California (*37*). HPS has been reported in other persons whose occupations entailed close physical contact with wild rodents (*38**,**39*).

The person in this study who was antibody-positive against WWAV had worked with rodents in Texas and handled woodrats. Antibodies against WWAV strain AV 9310135 have been found in southern plains woodrats (*Neotoma micropus*) captured in western Texas and in northern Texas (M.L. Milazzo, unpub. data), and arenaviruses antigenically closely related to WWAV strain AV 9310135 have been isolated from southern plains woodrats captured in southern Texas (*27**,**40*). Thus, the antibodies against WWAV in this person could be a result of an arenavirus infection acquired from a woodrat captured in Texas.

When examined by antibody-antigen binding assays such as the ELISA, GTOV is distinct from the 3 North American arenaviruses and highly cross-reactive with JUNV, MACV, and SABV (*19*). Thus, the antibodies against GTOV in the person in this study could be a result of an arenavirus infection acquired while traveling in South America. Alternatively, the antibodies could be a result of infection with a North American arenavirus that is antigenically more closely related to GTOV than to BCNV, TAMV, or WWAV.

Recently, antibodies against GTOV but not WWAV or LCMV were detected in 3 peromyscine rodents (*Peromyscus* species) captured in southern Mexico (M.L. Milazzo, unpub. data). The antibodies against GTOV in these 3 rodents are the first evidence that an arenavirus antigenically distinct from BCNV, TAMV, WWAV, and LCMV exists in North America and support the idea that the infection in the antibody-positive person in this study was a result of an arenavirus infection acquired in North America.
